# A non-expensive bidimensional kinematic balance assessment can detect early postural instability in people with Parkinson’s disease

**DOI:** 10.3389/fneur.2023.1243445

**Published:** 2023-11-17

**Authors:** Gabriel Venas Santos, Matheus Silva d'Alencar, Andre Frazão Helene, Antonio C. Roque, José Garcia Vivas Miranda, Maria Elisa Pimentel Piemonte

**Affiliations:** ^1^Department of Physical Therapy, Speech Therapy and Occupational Therapy, Faculty of Medical Science, University of São Paulo, São Paulo, Brazil; ^2^Department of Physiology, Institute of Biosciences, University of São Paulo, São Paulo, Brazil; ^3^Department of Physics, School of Philosophy, Sciences and Letters of Ribeirão Preto, University of São Paulo, Ribeirão Preto, Brazil; ^4^Institute of Physics, Laboratory of Biosystems, Federal University of Bahia, Salvador, Brazil

**Keywords:** Parkinson’s disease, balance, postural instability, cinematic assessment, early Parkinson

## Abstract

**Background:**

Postural instability is a debilitating cardinal symptom of Parkinson’s disease (PD). Its onset marks a pivotal milestone in PD when balance impairment results in disability in many activities of daily living. Early detection of postural instability by non-expensive tools that can be widely used in clinical practice is a key factor in the prevention of falls in widespread population and their negative consequences.

**Objective:**

This study aimed to investigate the effectiveness of a two-dimensional balance assessment to identify the decline in postural control associated with PD progression.

**Methods:**

This study recruited 55 people with PD, of which 37 were men. Eleven participants were in stage I, twenty-three in stage II, and twenty-one in stage III. According to the Hoehn and Yahr (H&Y) rating scale, three clinical balance tests (Timed Up and Go test, Balance Evaluation Systems Test, and Push and Release test) were carried out in addition to a static stance test recorded by a two-dimensional movement analysis software. Based on kinematic variables generated by the software, a Postural Instability Index (PII) was created, allowing a comparison between its results and those obtained by clinical tests.

**Results:**

There were differences between sociodemographic variables directly related to PD evolution. Although all tests were correlated with H&Y stages, only the PII was able to differentiate the first three stages of disease evolution (H&Y I and II: *p* = 0.03; H&Y I and III: *p* = 0.00001; H&Y II and III: *p* = 0.02). Other clinical tests were able to differentiate only people in the moderate PD stage (H&Y III).

**Conclusion:**

Based on the PII index, it was possible to differentiate the postural control decline among the first three stages of PD evolution. This study offers a promising possibility of a low-cost, early identification of subtle changes in postural control in people with PD in clinical practice.

## 1 Introduction

Balance impairment (BI), i.e., deficiency to control the body’s center of mass over its support’s base to achieve postural stability, is a common and debilitating motor alteration of Parkinson’s disease (PD), causing high disability levels (1–3). BI is a remarkable signal of postural instability, a complex and poorly understood motor symptom identified as a feature of PD in its late stages (4). Postural instability occurs 10–15 years after first diagnosis (5) and can be clinically detected from moderate disease stages, being the cardinal signal for progression from stage II to stage III according to Hoehn and Yahr (H&Y) rating scale, the most commonly used scale to control PD evolution (6). Despite the severe consequences involving BI, imposing an increased fall risk as the disease progresses, only 2% of newly diagnosed people with PD (PwPD) were classified as fallers and 15% as rare fallers (7), pointing to a necessary more attentive view of the issue.

BI is an independent risk factor for falls, injury, and significant mobility restriction, with a high negative impact on functionality. Unfortunately, the responsiveness of postural instability to current treatment strategies is limited (2, 3). Regarding medication treatment, although some significant positive effects are reported, there is no consensus regarding the effects of Levodopa on balance (8). Regarding surgical treatment, according to the target neural structure, deep brain stimulation may decrease or improve the balance in PwPD (9). Regarding non-medication treatment, a compelling review (10) demonstrates that exercise intervention reduces the rate of falls on PwPD, albeit with a modest effect.

Changes in BI are deeply related to PD. In healthy people, postural responses to perturbations are generated and controlled by automatic mechanisms that maintain standing posture and prevent falls (11). The sequence of events is automatically triggered in response to postural perturbations: (1) activation of sensory systems; (2) integration of sensory information; and (3) planning an adequate motor response to maintain the body’s center of gravity within the base of support (12). Nigrostriatal dopaminergic denervation and white matter alterations (13) may affect this ability in at least three distinct ways: (1) by impairing proper sensory integration involving the basal ganglia; (2) by perturbing the adjustment process for an appropriate escalating neuromuscular response; and (3) by perturbing the adjustment of muscle tone (14). Furthermore, non-motor aspects, mainly cognitive impairment in PD, such as altered attention, narrow cognitive focus, comorbid dementia, and fear of falls, could also affect balance control (15).

Early postural instability detection is an important challenge in PD since it can be used to diagnose and categorize PwPD in severity stages and subtypes based on phenotypes, i.e., tremor and axial (16). Furthermore, early postural instability detection is essential to identify people with increased fall risk. It must include accurate, time- and cost-effective assessments to identify patients at high risk of falling to allow timely preventive intervention (17). Adequate and timely recognition of balance disorders is critical to avoid injuries associated with falls, worsening quality of life, reduced mobility, and social isolation. In addition, quantifying balance deficits is relevant for monitoring patients over time (18).

It is widely recognized that PD includes BI and a consequent increased risk of falls as the disease progresses. However, there is a trend toward underreporting of BI (7). Given these concerns, clinical and laboratory instruments are developed to assess the postural instability associated with PD. These assessments can play a crucial role in objectively measuring and monitoring balance problems faced by individuals with PD in a controlled environment.

The efficient clinical evaluation of postural control and balance is crucial to guide the intervention to preserve functionality and decrease the fall risk in PwPD. Several clinical tests, such as the Berg Balance Scale (BBS), Tinetti, Mini-Balance Evaluation Systems Test (Mini-BESTest), Timed Up and Go (TUG) test, and Pull-test (PT), have been used to evaluate the balance and the postural control in PwPD (18). Scales based on the self-perception of BI have also been used to identify the fall risk in PwPD (19). The main advantages of this kind of test and scale are the short time and ease of application, no demand for sophisticated equipment, and, consequently, the low cost. On the other hand, results obtained by self-perception are subjective and may be biased by cognitive and mood alterations, which are common in PwPD. Clinical tests depend on the personal and subjective interpretation of the examiner and cannot offer a detailed and precise quantification and qualification of balance alterations (20).

A review of the psychometric properties of balance and fall risk prediction measures in PD showed that only 6 of the 68 outcome measures have strong psychometric properties. Among them, the Mini-BESTest and Push and Release test are best at body level (21). Furthermore, a critical review by the International Parkinson and Movement Disorders Society Task Force assessed the clinometric properties of existing rating scales, questionnaires, and timed tests that assess gait, balance, and posture alterations in PD. They found no scale suitable for evaluating gait, balance, and posture, as none of the instruments investigated adequately or separately assessed all constructs (18).

Besides the clinical tests, several measures to assess balance and fall risk prediction that require the use of laboratories or sophisticated instruments have been developed. These instruments assessed the ability to shift the mass and gravity center, spatiotemporal gait parameters, and sensory integration to quantify balance and/or fall risk (21). “Posturography,” “wearable devices,” “gait analysis,” and “center of pressure” (COP) have been used to track the postural control and gait of PwPD. Studies using posturography showed that people in the early stages of PD have a decrease in the limit of stability area and an increase in postural sway, and these conditions gradually deteriorate as the disease progresses (22, 23). Early abnormalities of anticipatory postural adjustments during turning in individuals in H&Y stage II (24) and abnormal standing sway in newly diagnosed individuals have also been demonstrated (25). However, a recent study showed that static posturography could detect significant balance decline only between very early and intermediate stages of disease progression, i.e., between H&Y stages I and III (16). Wearable devices installed in the neck, waist, back, lower limbs, and other body parts can also detect subtle changes in postural instability and the fall risk of PwPD (26).

Although these laboratory-based instruments may be used as an objective complementary tool to clinical balance tests to assess balance performance in PwPD, sophisticated tools to evaluate balance and fall risk have limited clinical utility because they are expensive. Such instrumentation is only commonly available in some clinics. Thus, clinic-based or bedside assessments using this equipment on a routine basis are only eventually possible.

Postural sway is a sensitive measure of the complex sensorimotor control loop responsible for controlling standing balance; it has been considered an excellent measure of postural instability (27). Traditionally, postural sway has been measured with a force plate under the feet. However, Ciria et al. (28) recently demonstrated that results obtained by two-dimensional kinematic evaluation of the head movements during stance posture were strongly correlated and coherent with COP sway registered by the force platform. Therefore, measuring head movements can be an alternative for studying human postural changes.

Recently, a new approach to movement analysis based on movement decomposing has been proposed by Miranda et al. (29) This method allows for a more detailed analysis of movement kinematics, providing a nonlinear approach to motor control characteristics. Considering the complex changes in movement in PwPD, this approach may be helpful. In fact, using this new approach, D’Alencar et al. (30) showed that the index provided by a two-dimensional movement analysis that uses kinematic gait variables was more sensitive to detect subtle gait alterations in early PD stages than clinical tests. Therefore, a similar method based on two-dimensional kinematic analysis of head movements during stance posture may also offer a non-expensive clinic-based evaluation that is more objective when compared to current recommended clinical tests to identify changes in postural control in PwPD. This method could be used isolated or combined with clinical tests to identify the progression of postural instability in PwPD. Recent studies have shown that models combining clinical and inertial sensor outcomes showed higher discriminative ability in classifying fallers and non-fallers among PwPD than clinical-only or mobility-only models (31, 32).

While new methods can provide a more objective evaluation of balance disruption and fall risks, their clinical utility could be limited due to costs, team, and equipment requirements. Recognizing this limitation, the primary objective of this study was to explore a straightforward and cost-effective approach that holds potential for clinical application in identifying the progression of postural instability in individuals with PD. By developing a more accessible method, the study aimed to enhance the practicality and feasibility of assessing postural instability in a clinical setting based on a two-dimensional kinematic evaluation of the balance performance in PwPD.

## 2 Materials and methods

### 2.1 Participants

A convenient sample of 55 people with PD (above 50 to allow for high-quality estimates according to COSMIN standards), 37 men, 11 participants in stage I, 23 in stage II, and 21 in stage III according to the H&Y rating scale were recruited from Brazil’s AMPARO Network.^1^ The study included individuals who met the following criteria: (1) They had idiopathic PD (stages I–III according to the H&Y rating scale) diagnosed by an experienced specialist in movement disorders using the UK Brain Bank criteria (33), and they were taking antiparkinsonian medications; (2) They were capable of independent ambulation; and (3) They showed no signs of dementia (determined by a Montreal Cognitive Assessment [MoCA] score above 21) or major depression (determined by a Geriatric Depression Scale score below 6). Additionally, participants were excluded if respiratory or cardiovascular diseases, clinically significant musculoskeletal alterations, other neurological disorders, or uncorrected visual/auditory impairments were present.

### 2.2 Design and procedures

**Figure 1:**
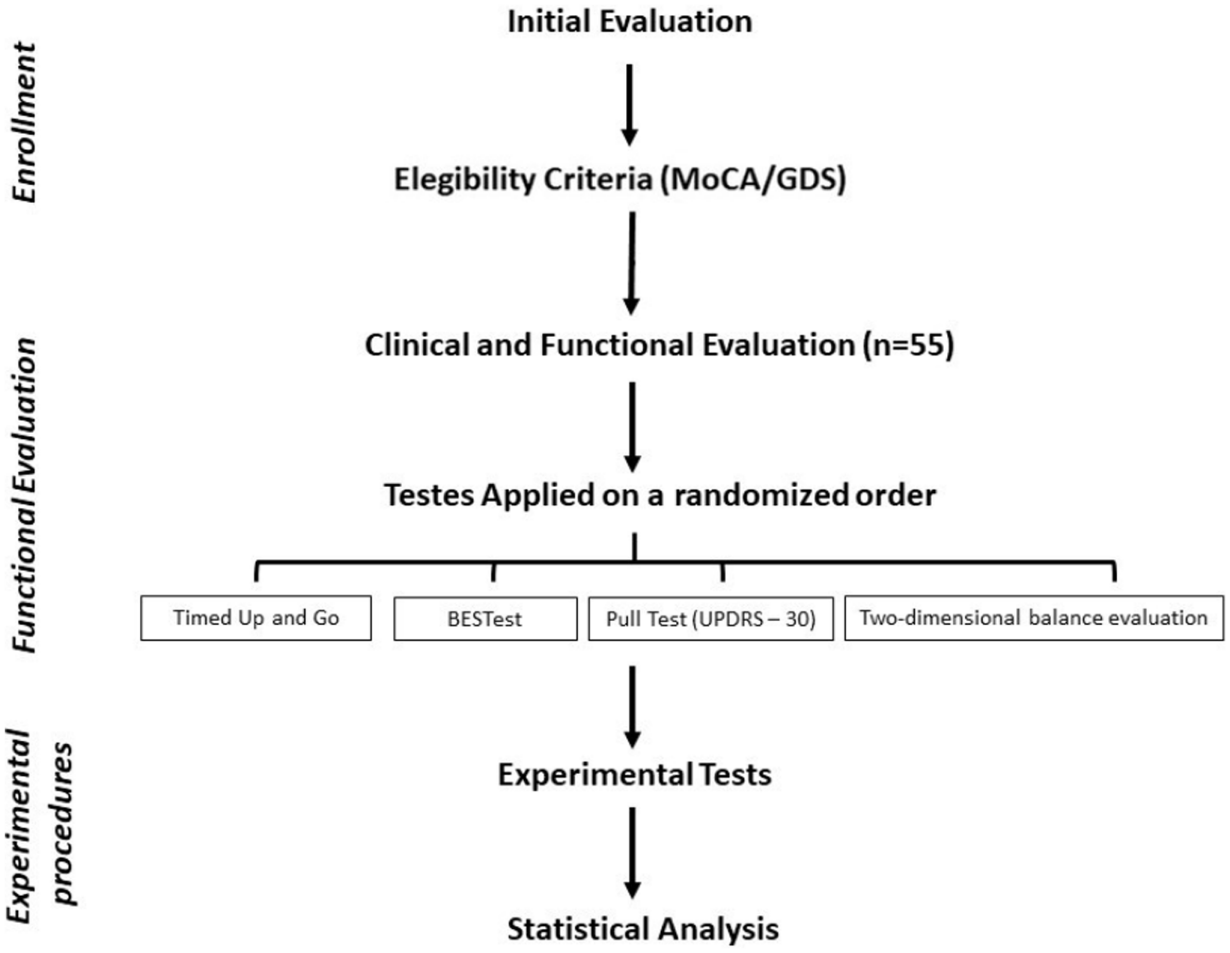
Flowchart of the study steps and procedures.

The present study obtained approval from a local ethics committee (#CAAE 67388816.2.0000.0065) and adhered to the principles outlined in the Declaration of Helsinki. Prior to the commencement of the study, each participant provided written informed consent. A cross-sectional design was employed, wherein participants underwent both motor and cognitive evaluations within a single session. These assessments were carried out by a physiotherapist with specialized expertise in movement disorders. All individuals diagnosed with PD were tested during their ON period, which occurred 40 to 120 min after their L-dopa dose. A detailed overview of the study’s stages and procedures is presented in Figure 1.

#### 2.2.1 TUG test

The TUG test is an easy, inexpensive, and efficient clinical application to assess mobility and functional balance. Participants were instructed to get up from a chair and walk in a straight line at their normal speed for 3 m, walk around a marked area, and then go back to the chair and sit down. The procedure was timed in seconds, starting with the command to do the test and the moment when the participant gets up from the chair until the participant returns to the chair and sits. The use of supports, crutches, and canes to help them was not allowed.

This measurement is useful in an outpatient setting because it only requires a few minutes and easy-to-handle equipment. The TUG test is shown to be highly correlated with functional mobility and gait speed in PD (34) and has also been proven to have high test–retest reliability and inter-rater reliability in PD (35).

#### 2.2.2 Pull test: unified Parkinson disease rating scale – Section III

The 30-item Push and Release test of Section III of the Movement Disorder Society-sponsored Unified Parkinson’s Disease Rating Scale (MDS-UPDRS) was administered to assess BI. A satisfactory response to the pull test requires the ability to mount an adequately sized backward step to compensate for the rapid backward displacement in the center of gravity initiated by pulling backward on a patient’s shoulders (36).

This test was treated as a continuous variable scored on an ordinal severity scale from low (0) to high (4). Excellent factor validity, test–retest reliability (ICC ¼ 0.93), high internal consistency, and responsiveness have been demonstrated (37). We proceeded according to the recommendation by Visser et al., i.e., a sudden, firm, rapid shoulder jerk without warning but with prior explanation and performed only once. Participants remained in a comfortable position with their eyes open, and the examiners stood behind the subjects, who were instructed to push back against the examiners’ palms placed on their shoulder blades while the examiners flexed the curves to allow for backward movement of the trunk while supporting the participants’ weight with their hands.

The examiners suddenly removed their hands when the subjects’ shoulders and hips moved into a stable position just behind their heels, allowing them to step back to regain balance.

#### 2.2.3 Balance evaluation systems test

The balanced performance of the participants in the groups was evaluated using the BESTest. The assessment process consists of six domains: biomechanical constraints, stability limits, anticipatory postural adjustments, postural response to the induced loss of balance, sensory orientation, and stability in gait (38). To use the BESTest to differentiate balance deficits, the examiner scores each item from 0 (worst) to 3 (best). The sum of all scores is the total result. Each category establishes its own result, making it very useful to know which postural control disorders are compromised. All evaluators were previously trained to apply the test.

#### 2.2.4 Two-dimensional balance analysis

The two-dimensional balance assessment was performed using the following instruments:

– A GoPro™ Hero Silver camera
– A headband
– Yellow stickers measuring 19 mm in diameter
– Calibration paper featuring two reference points placed 20 cm apart
– Camera tripods with height adjustment (quantity: 01)
– The GoPro™ Hero 7 Black application
– CvMob™ software, version 3.6 (accessible at http://cvmob.ufba.br).

**Figure 2:**
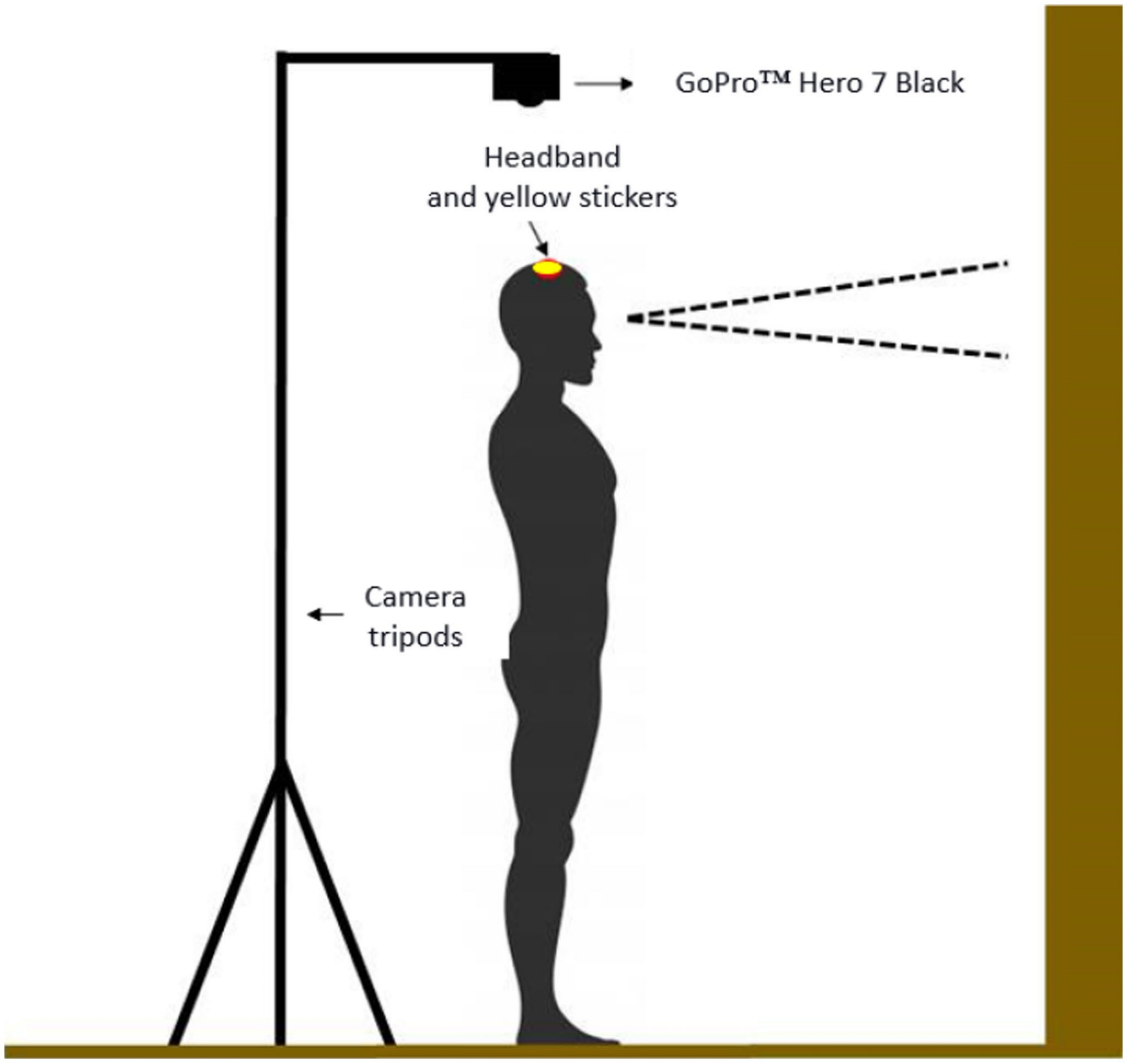
The representation of two-dimensional balance assessment procedures.

The participants were instructed to maintain a bipedal posture for 30 s from an auditory signal (GO), with their feet parallel (20 cm between them), on yellow dots marked on the floor. Their visual focus had to be held at a point located on the front wall. The height of the point in relation to the floor was adjusted according to the participant’s height. The camera was inserted in its silicone shield and fitted to a fixed adjustable tripod, being lowered and positioned at a distance of 20 cm from the reference point (yellow stickers) so that it recorded the top of the head (Figure 2).

The video parameters used for filming had the following configuration: (1) wireless control (connected to a Motorola™ Moto X Style smartphone); (2) narrow field of view; (3) 30 frames per second; (4) 720 bpi resolution; and (5) low light option turned off.

The kinematic balance variables were measured with the CvMob™ movement analysis system (39). From the trajectory and velocity data of the selected marker (sticker), the movement element decomposition method (29) was used to segment the movement into elements, defined by start and end at zero velocity. For each element, the method estimates the average velocity and displacement, and for the overall movement, it calculates the average displacement and the average of the average velocities of the elements, as well as the total quantity of elements found. These indices are estimated for each coordinate axis (anteroposterior and mediolateral). Only variables related to anteroposterior oscillation motion were used.

For constructing the Postural Instability Index (PII), three physiotherapists specialized in PD and a physicist specialized in movement analysis analyzed the behavior of all variables acquired by the CvMob™ system and their relationship with the evolution of PD. The behavior of the balance variables was analyzed to form an estimated value that directly correlates with postural stability, as inferred from the variability of oscillations in trajectory and velocity indicators. This estimated value has the potential to accurately reflect the progressive clinical evolution of the disease while considering the varying severity degrees. Based on the results of this exploratory analysis, we decided to include in the formula to calculate the PII only the three more powerful variables to detect the progression in postural instability in order to facilitate the implementation and interpretation in clinical practice.

The variables used in the formula were as follows: $\bar{V_{y}}$ : average velocity of the element size in the anteroposterior direction, which indicates the oscillation’s speed, which has been strongly associated with postural instability (40) and fall risk (21) in PD; *N_y_*: number of elements on the y-axis (antero-posterior), which indicates the number of inversions made in the oscillation’s trajectory; and $\bar{Dm_{y}}$ : average displacement size of the movement element in the y-direction (antero-posterior), which indicates the oscillation’s amplitude. Using these variables, the *PII* was defined according to Eq. (1):


(1) $$PII=\frac{\bar{V_{y}}.N_{y}}{\bar{Dm_{y}}}$$

Thus, the frequency and magnitude of oscillations play a central role in determining one’s postural stability, with a higher frequency of small oscillations in the anteroposterior direction resulting in decreased stability.

### 2.3 Analysis

Normal distribution of the samples was assessed using the Kolmogorov-Shapiro test, and for variables that demonstrated a normal distribution, such as age and MDS-UPDRS-III, the distribution homogeneity was tested using Levene’s test.

Variables did exhibit a normal distribution, including age, schooling, MoCA, UPDRS II, UPDRS III, TUG and BESTest, which were tested using one-way ANOVA, with the H&Y stages being considered as factors. The effect size was tested for each factor that reached a statistically significant level. In cases where statistically significant differences were detected, Tukey’s post-test was applied for pairwise comparisons between the groups.

Variables that did not exhibit a normal distribution, including GDS, MDS-UPDRS-30, and PII, were tested using Kruskal-Wallis ANOVA (KW-ANOVA), with the H&Y stages being considered as factors. When statistically significant differences were observed, multiple comparisons of the average ranks for each pair of groups were applied; normal *z*-values were computed for each comparison, as well as *post-hoc* probabilities (corrected for the number of comparisons) for a two-sided test of significance.

Additionally, Spearman’s rank-order correlation was used to test correlations between the balance measures and H&Y and MDS-UPDRS-III scores. Finally, the same test was used to test the correlation between PII and the other balance tests.

A significance level of *p* < 0.05 was used to determine the statistical significance of the findings. All statistical analyses were performed using Statistica version 13 (TIBCO Software Inc., United States).

## 3 Results

No significant differences were found among the groups in terms of age, gender, schooling, and GDS scores. However, as expected due to disease progression, significant differences were observed in MoCA, MDS-UPDRS-II, and MDS-UPDRS-III scores (Table 1). All balance measures showed significant correlations with H&Y stages and MDS-UPDRS-III (Table 2).

**Table 1:** Clinical and demographic characteristics of participants (*n* = 55).

Variable	H&Y1 (*n* = 11)	H&Y2 (*n* = 23)	H&Y3 (*n* = 21)	H/F	*p*-value	ES	H&Y1 vs. H&Y2	H&Y1 vs. H&Y3	H&Y2 vs. H&Y3
Age (Years)	65.4 (8.09)	66.3 (8.3)	68.5 (7.6)	0.64	>0.05^a^	–	–	–	–
Gender (Male)	7	16	13	–	>0.05^b^	–	–	–	–
Schooling (Years)	13.8 (4.4)	11.6 (4.02)	12.8 (5.7)	0.47	>0.05^a^	–	–	–	
MDS-UPDRS II (Score)	8.18 (3.7)	12.6 (3.7)	14.4 (7.2)	5.1	0.0003^a^	0.79	0.013^c^	0.003^c^	>0.05^c^
MDS-UPDRS III (score)	10.1 (4.5)	22 (7.3)	27.2 (12)	12.76	0.0001^a^	0.99	0.001^c^	0.001^c^	>0.05^c^
MoCA	26 (2.9)	25.2 (3.7)	23.7 (2.2)	4.18	0.0128^a^	0.71	>0.05^c^	0.015^c^	>0.05^c^
GDS	2.3 (1.5)	3.3 (2.2)	4.3 (3.1)	4.14	>0.05^b^	–	–	–	–

For continuous variables, mean values are presented together with standard deviation values, in parentheses. H&Y, Hoehn and Yahr scale; UPDRS-III, Section 3 of the unified Parkinson’s disease rating scale; MoCA, montreal cognitive assessment; GDS, geriatric depression scale; ES, effect size.

a ANOVA one-way.

b Kruskal-Wallis ANOVA.

c Tukey post-test.

**Table 2:** Correlation between balance tests and H&Y stages.

H&Y stage	Spearman R	*p*-value
Pull-test	0.83	0.000001
BESTest	−0.61	0.000001
TUG	0.53	0.000029
PII	0.80	0.000001

H&Y, Hoen & Yarh scale; Pull test, unified Parkinson disease rating scale– Section III; BESTest, balance evaluation systems test; TUG, timed up & go test; PII, postural instability index.

### 3.1 Pull-test and balance performance

**Figure 3:**
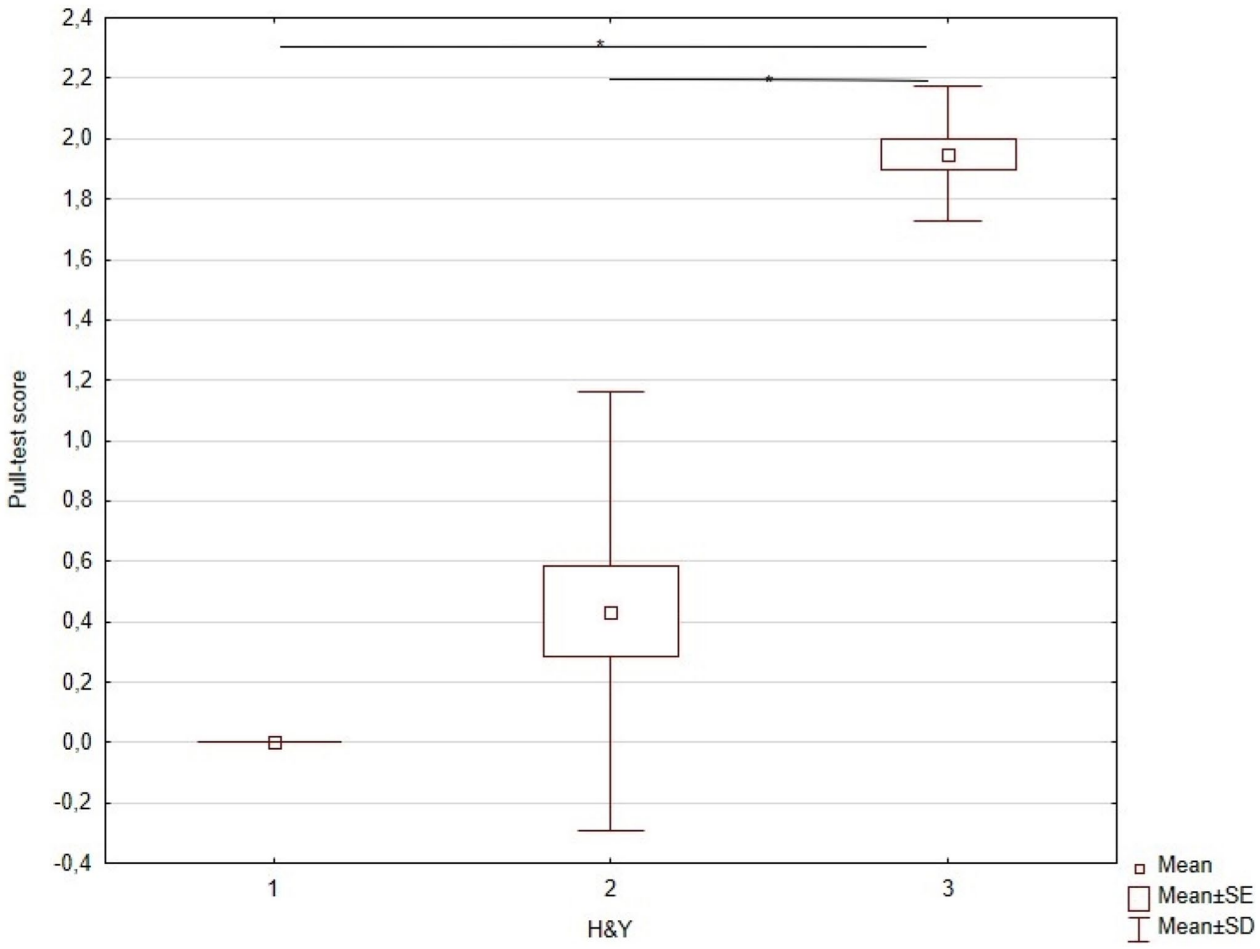
KW-ANOVA demonstrating the pull-test differences between H&Y stages I and III, and II and III.

The KW-ANOVA for test 30 of the MDS-UPDRS revealed a statistically significant effect of disease stages according to H&Y (Table 3). Subsequent multiple comparison tests indicated significant differences between stages I and III and stages II and III (Table 3), but no significant difference between stages I and II (Figure 3).

**Table 3:** Balance measures of participants (*n* = 55).

Variable	H&Y1 (*n* = 11)	H&Y2 (*n* = 23)	H&Y3 (*n* = 21)	H/F	*p*-value	ES	H&Y1 vs. H&Y2	H&Y1 vs. H&Y3	H&Y2 vs. H&Y3
Pull-test	0.00 (0.0)	0.43 (0.72)	1.95 (0.22)	36.37	0.00001^b^	–	>0.05^d^	0.00003^d^	0.00001^d^
BESTest	89.72 (4.96)	82.69 (7.35)	73.85 (9.89)	15.12	0.00001^a^	0.99	>0.05^c^	0.001^c^	0.001^c^
TUG	7.58 (1.61)	9.06 (1.59)	11.20 (2.71)	11.93	0.000^a^	0.99	>0.05^c^	0.001^c^	0.004^c^
PII	0.46 (0.06)	0.69 (0.56)	1.27 (0.62)	33.21	0.00001^b^	–	0.0006^d^	0.0002^d^	0.000001^d^

For continuous variables, mean values are presented together with standard deviation values, in parentheses. H&Y, Hoen & Yarh scale; Pull test, unified Parkinson disease rating scale– Section III; BESTest, balance evaluation systems test; TUG, timed up & go test; PII, postural instability index; ES, effect size.

a ANOVA one-way.

b Kruskal-Wallis ANOVA.

c Tukey post-test.

d Kruskal-Wallis multiple comparisons.

### 3.2 TUG and balance

**Figure 4:**
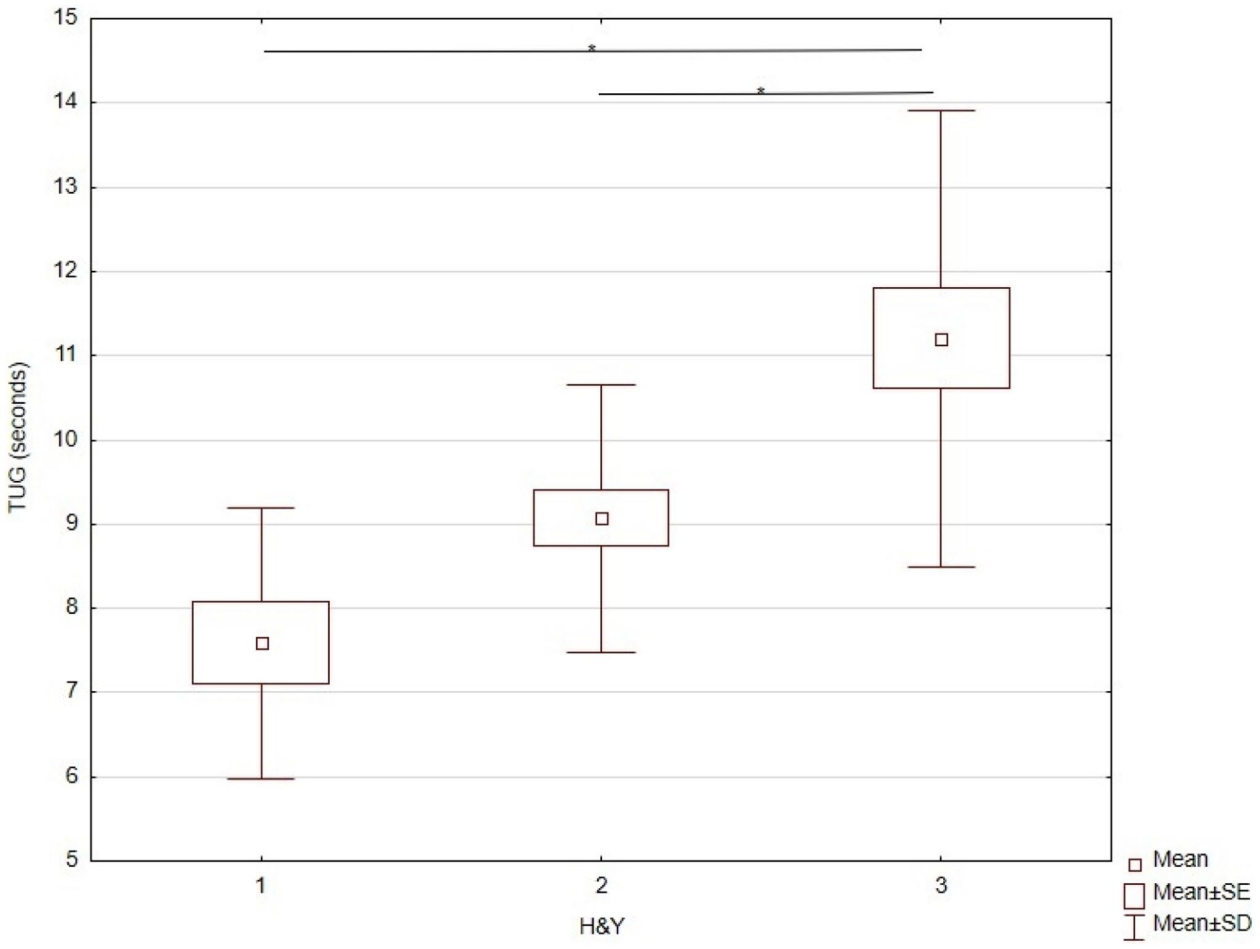
KW-ANOVA demonstrating TUG differences in H&Y stages between I and III, and II and III.

The one-way ANOVA for the time taken to complete the TUG test demonstrated a statistically significant effect of disease stages according to H&Y (Table 3). Tukey’s *post-hoc* test revealed significant differences between stages I and III and stages II and III (Table 3), but no significant difference between stages I and II (Figure 4).

### 3.3 BESTest and balance performance

**Figure 5:**
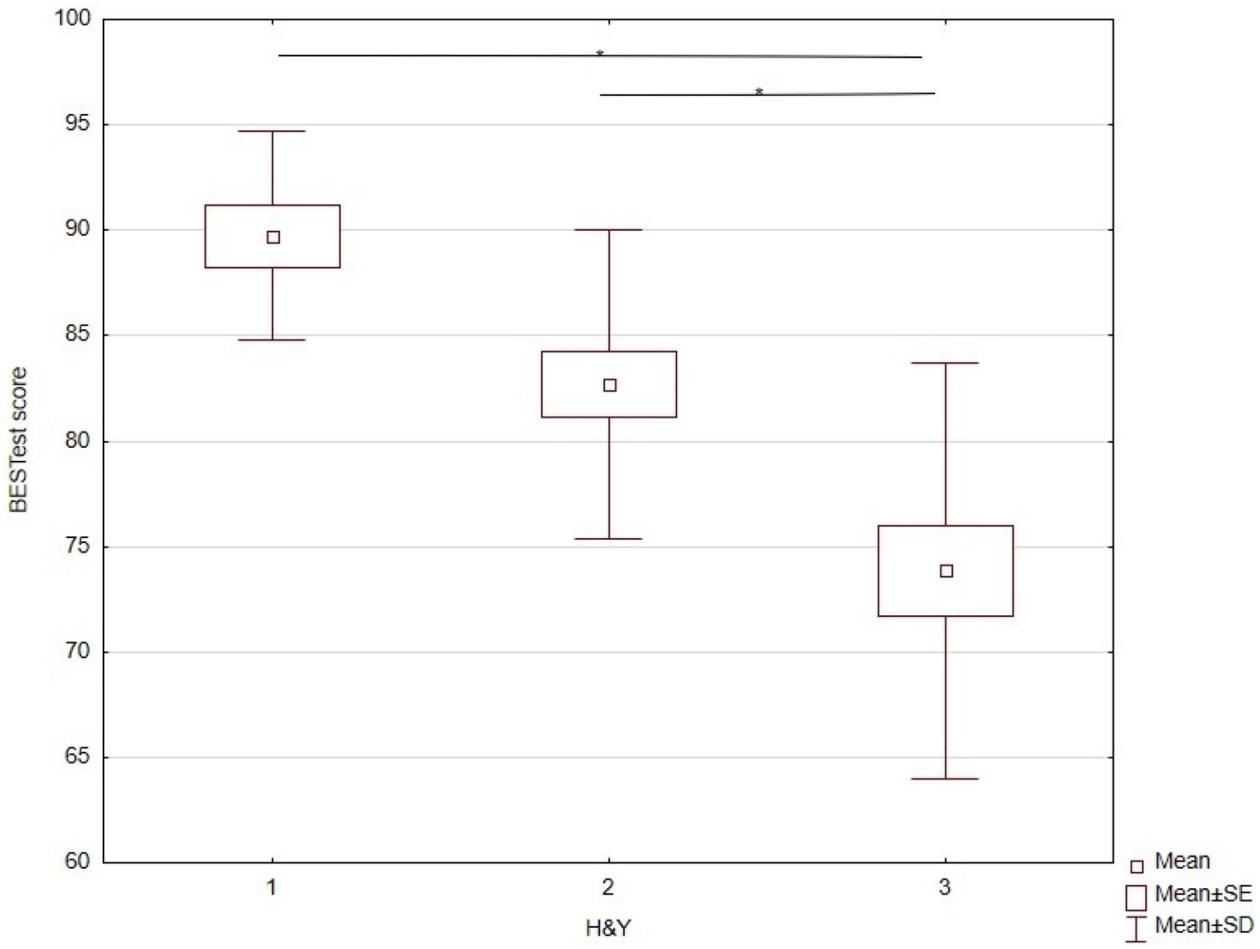
KW-ANOVA demonstrating the BESTest differences Between H&Y stages I and III, and II and III.

The one-way ANOVA for DGI scores showed a statistically significant effect of disease stages according to H&Y (Table 3). Tukey’s *post-hoc* test indicated significant differences between stages I and III and stages II and III (Table 3), but no significant difference between stages I and II (Figure 5).

### 3.4 Two-dimensional balance evaluation

**Figure 6:**
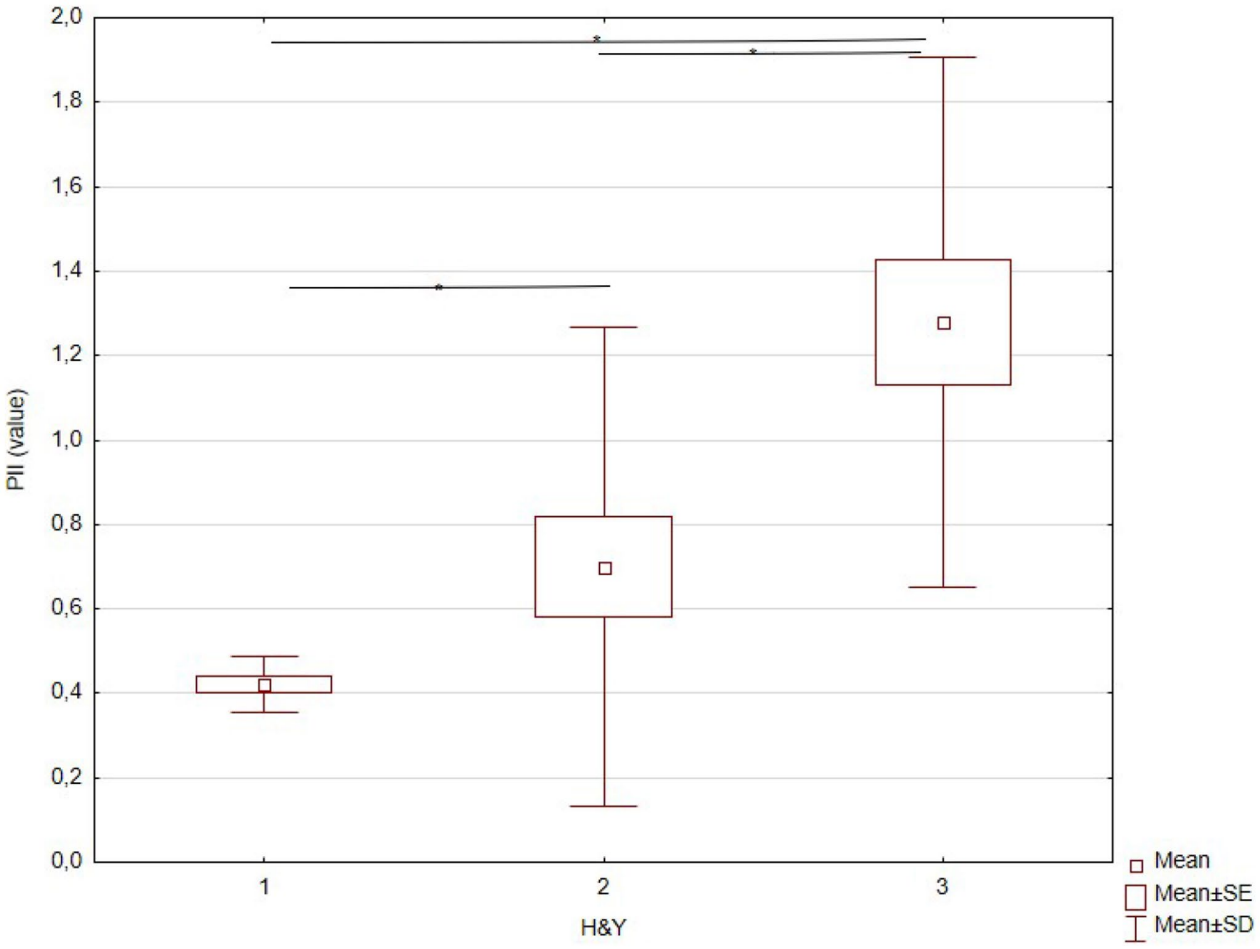
KW-ANOVA demonstrating the PII differences between H&Y stages I and II, II and III, and I and III.

The KW-ANOVA for the PII revealed a statistically significant effect of disease stages according to H&Y (Table 3). Multiple comparisons indicated significant differences between stages I and II (*p* < 0.006), stages I and III, and stages II and III (Table 3; Figure 6).

Furthermore, the PII was statistically significantly correlated with all clinical balance tests, as can be observed in Table 4.

**Table 4:** Correlation between PII and balance tests.

PII	Speaman R	*p*-value
Pull-test	0.70	0.000001
BESTest	−0.53	0.000001
TUG	0.54	0.00001

Pull test, unified Parkinson disease rating scale– Section III; BESTest, balance evaluation systems test; TUG, timed up & go test; PII, postural instability index.

## 4 Discussion

Several laboratory-based instruments have been used to evaluate the postural instability associated with PD. Although they may offer a more objective evaluation than clinical tests, the use of sophisticated equipment to identify balance disruption and fall risks has limited clinical utility because they are expensive and demand a highly trained team. This study aimed to investigate a straightforward and non-expensive approach with potential clinical application to identify the progression of postural instability in PwPD.

Our results show that the PII, obtained through the proposed bidimensional kinematic evaluation of head movements during quiet posture, was effective in identifying the progressive increase in postural instability between H&Y stages I, II, and III of PD, while currently recommended clinical tests were able to show significant differences only between the initial and intermediate stages of the disease (I and III; II and III). In other words, the PII could identify subtle balance alterations between two early stages of disease progression.

In the past decade, the number of articles published on postural control in PwPD has increased annually in the scientific literature. This upward trend in article production over time suggests that postural control in PwPD has gradually gained importance as a research topic (20). Currently, the most common way to assess postural control in clinical practice is to use rating scales and motor tests. These tools are susceptible to clinician bias, are insensitive to mild impairments (ceiling effects), and have low reliability (41, 42). Our findings made a significant contribution by demonstrating that the low-cost and user-friendly kinematic assessment we proposed exhibited greater sensitivity in detecting early signs of postural control decline compared to other commonly used clinical tests. This suggests that our assessment has the potential to be readily employed in clinical practice.

Postural instability in PwPD is correlated with the disease severity, being more pronounced in people in the more advanced stages of the disease (43). The progression from H&Y stage II to III marks a critical milestone in PD when gait and BI result in increased motor disability, reducing independence in daily living activities (44). In fact, in the present study, all adopted clinical tests were correlated with H&Y stages and could detect progression in the postural instability from I or II to III H&Y stages. A longitudinal study with PwPD showed that a balance deficit is observed in up to 70% of people in the advanced stages of the disease, being one of the main risk factors for falls (45). Other studies using clinical tests (37) or several different technologies, such as force platforms to measure the COP displacement during quiet posture (46, 47), posturography to evaluate postural sway (48), and accelerometry (25) to measure the range of motion variability, average movement velocity, and movement asymmetry (49, 50), have shown increased postural instability from moderate to advanced PD stages.

Current understanding of postural control changes in early to moderate PD is limited and requires further clarification. Few studies have compared or correlated the balance changes between disease stages and progression, especially the early ones (51). Duncan et al. (52) showed that balance performance measured by the BESTest declined over 6 to 12 months in PwPD. However, only four participants in this study were in H&Y stage I, and the BESTest score was only weakly correlated with the H&Y stages at the study beginning. A review of TUG’s psychometric and clinical properties indicated that the H&Y stage must be consistently recorded in the different studies. The authors recommended that further studies divide the sample into stages when performing balance analyses rather than just providing descriptive population data (53). One of the few studies that compared TUG performance in early PD stages (H&Y I and II) and controls found no difference, suggesting no balance decline in early disease stages (54). Finally, the PD severity could be tracked objectively by the quantifiable responses of the pull-test parameters (36), the more relevant alteration can be observed in people in H&Y stage III who had a significantly impaired compensatory response to backward pull (55). Then, it is not a surprise that in the present study, all tests mentioned above, despite being correlated with the H&Y stage, could distinguish only between stages III and I–II.

The studies using laboratory-based instruments have shown better sensitivity to detect postural instability in the early PD stage than clinical tests. Among the 32 studies included in a review on posturography to assess postural control in PD, only half of them included PwPD in H&Y stage I, and only some took into account the stage of the disease’s evolution in the analysis of results (56). Studies using this method showed that PwPD in the early stage had a larger sway area (57) and a larger anteroposterior and mediolateral sway range (58) than the control subjects. This previous study also showed that PwPD in H&Y stage II–III presented higher postural control asymmetry than in H&Y stage I. Mild baseline subclinical changes in postural sway were found in PwPD in H&Y stage I (only two participants) and II (59). Studies found a significant correlation between sway indices (22) and anteroposterior and mediolateral sway ranges recorded with eyes closed (60) and H&Y stages (I–III) but did not investigate differences among each H&Y stage. Low-frequency modulation of the center of the pressure may differentiate PwPD in H&Y stage II from those in stage III (56). Small perturbations can more easily destabilize PwPD in H&Y stage III than those in stages I–II, showing larger CoP displacements (61).

Furthermore, PwPD in H&Y stages II–III presented higher postural control asymmetry than those in stage I (62). In contrast, when comparing postural instability among the three early PD stages, a recent study using static posturography could detect a significant balance decline only between H&Y stages I and III (16). The PII detected significant differences among the three H&Y stages in the present study.

The PII building from the kinematic variable obtained by head movement during quiet posture was based on the relationship between speed, range, and the number of moving elements in the anteroposterior direction. In fact, previous studies showed that increased oscillation of the COP in the anteroposterior direction is a remarkable alteration in PwPD, associated with postural instability (40, 63, 64). Furthermore, the mean root square of anterior–posterior trunk acceleration while standing on foam with eyes open was included in the top 10 ranked models to identify PwPD fallers (32). Most importantly, the stability threshold in anterior and posterior directions may have already decreased in H&Y stage II (65), and the relationship between anteroposterior and lateral stability (larger anteroposterior than lateral oscillations) has been observed since H&Y stages I and II (66). The early alterations in anteroposterior stability, marked by a higher frequency of small movements in the anteroposterior direction, may explain why the frequency, speed, and size of this head movement element in this direction used to calculate PII were able to differentiate the first three PD stages. Notably, individuals in moderate stages of PD face difficulties in scaling their postural responses effectively (67) and exhibit shorter steps, requiring more steps to respond to pulls in the anteroposterior direction (68). The decline in the ability to select and execute appropriate reactive movements regarding direction, amplitude, and speed could lead to postural instability in PD. This decline may be explained by the overlap of several alterations associated with PD, such as axial rigidity, bradykinesia of postural responses, impaired sensory integration, and less automaticity of postural responses (15). Although PII was strongly correlated with recommended clinical tests, it was able to show subtle alterations in anteroposterior stability between very early and early stages (H&Y I and II) that were not detected by clinical tests.

The reproducibility of the variables derived from the two-dimensional software employed in creating the PII must be more explored, highlighting the need for further research utilizing similar or more sophisticated resources. Continuing this effort, our study has the potential to introduce a novel perspective for assessing subtle postural instability in the early stages of PD, facilitating early therapeutic interventions. Moreover, the PII assessment holds promise for detecting balance alterations both pre- and post-intervention, offering valuable, innovative, and non-expensive interventions.

A recent study has shown that the age of PwPD instead of disease duration defines the onset of postural instability, i.e., the older the PwPD at disease onset, the sooner the postural instability onset (69). The present study showed no significant difference between PwPD in H&Y stages I, II, and III. Then, the age differences cannot explain the current results.

Although the results of this study are reliable and significant, we should highlight some limitations. The foremost is the small number of participants, especially regarding PwPD early PD stage (H&Y stage I). More participants in this stage of PD should be analyzed in further studies to confirm our findings. However, considering that participants were strictly selected and the clinical and postural evaluations were performed according to gold-standard scientific procedures, including the randomization of the order of the tests’ application, the relevance of the study’s contribution is still maintained. Additionally, the PII was built based on head oscillations only. Although a previous study using an identical method with healthy people showed a high correlation between COP measurements obtained by the force platform and head movement obtained by CvMob (28), further studies should conduct a direct comparison with other state-of-the-art methods in PwPD.

By utilizing two-dimensional movement analysis software that incorporates kinematic postural variables, we successfully distinguished variations in BI across the initial three stages of PD progression. This study presents a hopeful prospect of employing a clinical tool to detect subtle alterations in the postural control of individuals with PD.

## Data availability statement

The original contributions presented in the study are included in the article/supplementary material, further inquiries can be directed to the corresponding author.

## Ethics statement

The present study obtained approval from a Local Ethical Committee (#CAAE 67388816.2.0000.0065) and adhered to the principles outlined in the Helsinki Declaration. The studies were conducted in accordance with the local legislation and institutional requirements. The participants provided their written informed consent to participate in this study.

## Author contributions

GS: research project (organization and execution), statistical analysis (design), and manuscript (writing of the first draft). Md’A: research project (organization and execution) and manuscript (writing of the first draft). AH and AR: manuscript (review and critique) and statistical analysis (review and critique). JM: research project (conception), statistical analysis (review and critique), and manuscript (writing of the first draft, review, and critique). MP: research project (conception, organization, and supervision), statistical analysis (design, execution, review, and critique), and manuscript (writing of the first draft, review, and critique). All authors contributed to the article and approved the submitted version.

## Footnotes

^1^ www.amparo.numec.prp.usp.br
